# Synthesis of N-arylmethyl Substituted Indole Derivatives as New Antiplatelet Aggregation Agents 

**Published:** 2014

**Authors:** Masoud Faghih Akhlaghi, Salimeh Amidi, Marjan Esfahanizadeh, Marjan Daeihamed, Farzad Kobarfard

**Affiliations:** a *Department of Medicinal Chemistry, School of Pharmacy, Shahid Beheshti University of Medical Sciences, Iran, Vali Asr Ave, Niayesh Junction, Tehran, Iran.*; b *Phytochemistry Research Center, Shahid Beheshti University of Medical Sciences, Iran.*; c *Department of Pharmaceutics, School of Paharmacy, Shahid Beheshti University of Medical Sciences, Tehran, Iran. *

**Keywords:** 1-(substituted benzyl)-3-(phenylimino)indolin-2-one, Antiplatelet, Platelet aggregation, N-arylmethyl indole

## Abstract

A number of N-arylmethyl substituted indole derivatives have been synthesized and their effectiveness against ADP and arachidonic acid induced platelet aggregation in human plasma was determined. The desired compounds were synthesized by reacting the appropriate aniline derivative with isatin (or substituted isatin) to form the corresponding imine structures. The so formed compound was then activated using sodium hydride and reacted with the proper substituted benzyl halides. Among the tested compounds, derivatives 4a, 4c, 4d, 4f-i and 4k were the most potent compounds with satisfactory IC_50 _values (under 38.5 μM) for inhibition of platelet aggregation induced by arachidonic acid.

All indole derivatives without substitution on position 1 of the indole ring, exhibited either weaker activities or were not active at all.

## Introduction

Cardiovascular diseases have been the leading cause of death all over the world for many years (1, 2). The pivotal role of platelets in haemostasis has been linked to the pathogenesis of atherothrombotic disease. Activation of platelets at the site of an atherosclerotic plaque rupture, results in thrombus formation and subsequent vessel occlusion, causing ischemic diseases such as myocardial infarction, stroke, or peripheral artery disease (3, 4). Therefore antiplatelet therapy has a key role in the management of patients with thrombotic disorders (5, 6). Various antiplatelet agents with different mechanisms of action are currently available (7-9). Among them the most commonly used therapies include aspirin (that impairs thromboxane A2 synthesis by irreversibly inhibiting cyclo-oxygenase I), clopidogrel (an irreversible antagonist of platelet ADP (adenosine diphosphate) receptor, P2Y12), and glycoprotein (GP) IIb–IIIa antagonists like abciximab and eptifibatide (10, 11). In spite of their effectiveness, these agents suffer from some important limitations including limited efficacy, inter-individual variability and drug resistance, high risk of bleeding, or the need for parenteral administration (9, 10). It is predicted that by 2020, heart disease and stroke will become the leading cause of death and disability worldwide (12) and the shortcomings of current therapies call for development of safer and more effective antiplatelet agents.

**Figure 1:**
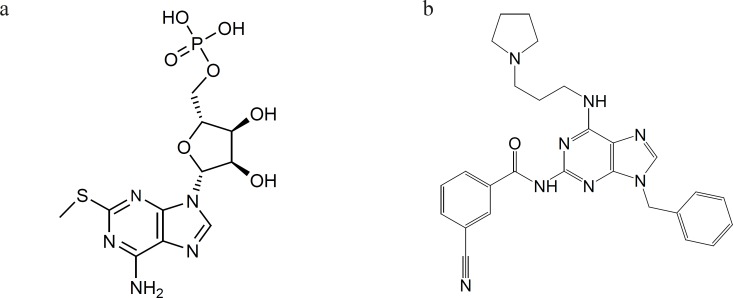
Compounds with similar structure to purine base that show antiplatelet activity. a: 2-methylthioadenosine 5›-monophosphate triethylammonium salt. b: 3-Cyano-N-[9-phenylmethyl-6-(3-(pyrrolidinyl)-propylamino)-9H-purin-2-yl] benzenecarboxamide semihydrate

Recent studies have shown that compounds with similar structure to purine base (Figure 1) are competitive ADP receptor antagonists (13, 14). 

Indole is a nonpolar purine analog (15) that is present in some important biochemical molecules such as tryptophan, serotonin and melatonin (16). Furthermore there are currently many indole containing drugs in the market (16, 17) and indole ring has attracted the attention of many medicinal chemists as an interesting scaffold in the process of new drug development (16, 18, 19). Therefor it could be rationally considered as an appropriate core for designing new antiplatelet agents. 

Studies of Marschenz and Rehse reveal the antiplatelet activity of N-(purin-2-yl) benzenecarboxamides (13). On the other hand Wu *et al. *reported antiplatelet activity of ethyl 4-(1-benzyl-1H-indazol-3-yl)benzoate derivatives (20). 

**Figure 2:**
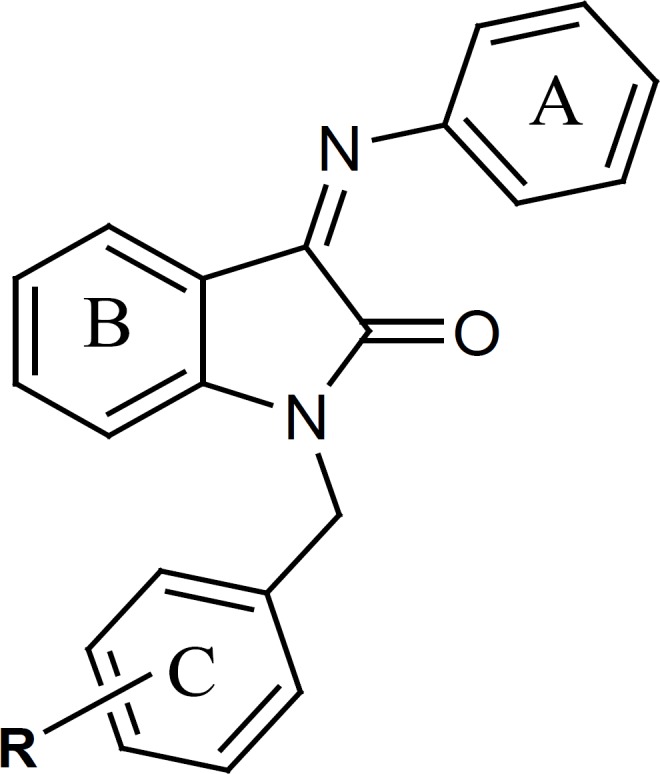
General structure of 1-(aryl)-3-(phenylimino)indolin- 2-one derivatives

The present study describes the synthesis of a group of N-(substituted benzyl) indole derivatives with an aryl imine structure on position 3 of indole ring. In order to study the structure-antiplatelet activity relationship of 1-(substitutedbenzyl)-3-(phenylimino)indoline- 2-one derivatives, different aromatic rings with divers physicochemical and electronic properties have been introduced as R group on the benzylic moiety of the indole derivatives. (Figure 2).

**Figure 3:**
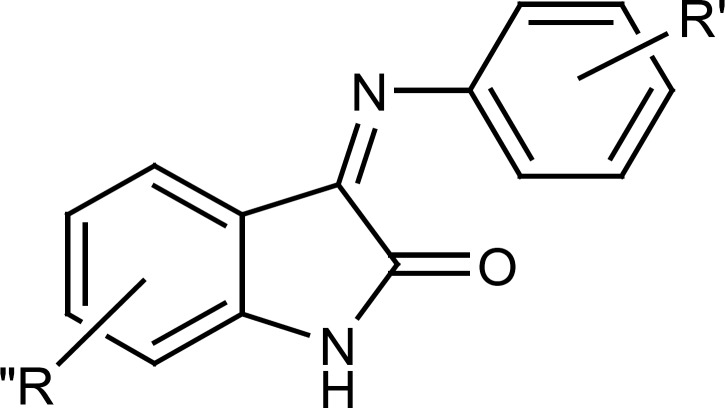
General structure of 3-(arylimino)indolin-2-one derivatives

Evaluation of antiplatelet activity of these derivatives and comparing their IC50 values with those of indole derivatives without the benzylic substituent, prompted us to synthesize a few other indole derivatives wich lacked the benzylic substituent on position 1 of indole ring but had different substituents either on pheylimino ring or on indole ring itself (Figure 3). 

Comparing the antiplatelet activity of these derivatives with those of the N-benzyl indole derivatives provided some insights about the role of benzyl substituent in exerting antiplatelet aggregation effects. 

## Experimental

Reactions were monitored by thin-layer chromatography (TLC) on silica gel (precoated F254 Merck plates) using ethyl acetate and n-hexan mixture as mobile phase. Melting points were measured by an Electrothermal 9100 apparatus and are uncorrected. The Infrared spectra (IR) were obtained on a Perkin-Elmer 843 spectrometer with KBr as diluent. Electrospray ionization mass spectra (ESI-MS) were obtained using Agilent 6410 Triple Quad mass spectrometer. Proton nuclear magnetic resonance (1H NMR) spectra were recorded in CDCl3 solution on a Bruker Avance DRX 500 MHz spectrometer. Peak positions are reported in parts per million (*δ*) downfield from tetramethylsilane as internal standard, and *J *values are given in hertz. All the compounds were analyzed for C, H, N and O on a Costech model 4010 and agreed with the proposed structures within ±0.4% of the theoretical values. 

*General procedure for the Synthesis of 3-(arylimino)indolin-2-one derivatives (3a-i)*

Isatin (7.36 g, 50 mmol) was added to 30 mL of ethanol solution containing 2 mL glacial acetic acid, and the solution was stirred at reflux temperature for 20 minute. The appropriate aniline derivative (50 mmol) was added to the so formed solution and the mixture was stirred at 100 0C for 3-20 hours (monitored by TLC). After completion of the reaction indicated by TLC, the developed precipitates were filtered off, washed with water (10–15 mL), dried in vacuum oven and recrystallized from appropriate solvents. Spectroscopic data for compounds 3a-i have been reported elsewhere (21). 

*General procedure for the Synthesis of 1-(aryl)-3-(phenylimino)indolin-2-one derivatives (4a-k)*

Into the solution of compound 3a (0.8 g, 3.36 mmol) in anhydrous DMF (6.5 mL), sodium hydride (0.16 g, 445 mmol) was added at room temperature. After 10 min, the appropriate arylmethyl halide (3.9 mmol) was added rapidly and the mixture was stirred for 15 min. The solution was then poured into cold saturated NaCl solution while swirling and a bright orange precipitate formed. The precipitate was collected by vacuum filtration and subsequently washed with H2O, dried in vacuum oven and recrystallized from appropriate solvent. 

*Structural data*

1-(benzyl)-3-(phenylimino)indole-2-one (4a)

M.P. 118-120 oC. Yield: 62%. IR (KBr): 1732(CO), 1607,1506,1462 (Aromatic zone), 1348, 1205. MS(ESI): 313 (M+H+). 1H-NMR(500 MHz, CDCl3): 7.47-7.02 (10H; m; A,B, C rings), 6.78-6-61 (4H; m; C ring); Anal. Calcd. for C21H16N2O (312.36): C,80.75; H, 5.16; N, 8.97; O, 5.12. Found: C,80.72; H, 5.18; N, 8.95; O, 5.15. 

*1-(4-fluorobenzyl)-3-(phenylimino)indole- 2-one (4b)*

M.P. 148-151 oC. Yield: 65.88%. IR (KBr): 1734(CO), 1605,1506,1461 (Aromatic zone), 1350, 1215, 765. MS(ESI): 331 (M+H+), 353 (M+Na+). 1H-NMR(500 MHz, CDCl3): 7.48 (2H; t; J= 7.7 Hz; H-4 and H-7 of B ring), 7.40 (2H; t; J= 7.4 Hz; H-2 and H-6 of C ring), 7.30 (1H; m; H-6 of B ring), 7.1 (5H; m; H-2 and H-3 and H-4 and H-5 and H-6 of A ring), 6.8 (2H; d; J= 7.8 Hz; H-3 and H-5 of C ring), 6.7 (1H; d; J= 7.4 Hz; H-5 of B ring), 5 (2H; S; CH2). Anal. Calcd. for C21H15FN2O (330.36): C,76.35; H, 4.58; F, 5.75; N, 8.48; O, 4.84. Found: C,76.32; H, 4.59; F, 5.77; N, 8.45; O, 4.86.

*1-(3-fluorobenzyl)-3-(phenylimino)indole-2-one (4c)*

M.P. 136-139 oC. Yield: 63.17%. IR (KBr): 1737 (CO), 1613,1590,1466 (Aromatic zone),1353, 1106,767. MS (ESI): 331 (M+H+). 1H-NMR(500 MHz, CDCl3): 7.45 (2H; t; J= 8.2 Hz; H-5 and H-6 of B ring), 7.32 (1H; m; H-5 of C ring), 7.25 (2H; m; H-3 and H-5 of A ring), 7.16 (1H; d; J= 7.6 Hz; H-4 of B ring), 7.02 (2H; d; J= 8.4 Hz; H-2 and H-6 of A ring), 6.9 (1H, dt, J= 8.7 Hz; J= 2.6 Hz; H-4 of A ring), 6.75 (1H; d; J= 7.6 Hz; H-6 of C ring), 6.71 (1H; d; J= 8.1 Hz; H-2 of C ring), 6.65 (1H; d; J= 7.7 Hz; H-7 of B ring), 5 (2H; S; CH2); (Mixed isomers). Anal. Calcd. for C21H15FN2O (330.36): C,76.35; H, 4.58; F, 5.75; N, 8.48; O, 4.84. Found: C,76.34; H, 4.60; F, 5.73; N, 8.49; O, 4.85.

*1-(2-fluorobenzyl)-3-(phenylimino)indole-2-one (4d)*

M.P. 128-130 oC. Yield: 64.62%. IR (KBr): 1750 (CO), 1623,1477 (Aromatic zone), 1363, 1179, 861, 780. MS(ESI): 331 (M+H+). 1H-NMR(500 MHz, CDCl3): 7.5 (3H; m; H-4 and H-5 and H-6 of B ring), 7.3 (3H; m; H-3 and H-4 and H-5 of A ring), 7.15 (2H; m; H-4 and H-5 of C ring), 7.06 (2H; d; J= 7.6 Hz; H-2 and H-6 of A ring); 6.9 (1H; d; J= 7.9 Hz; H-6 of C ring); 6.8 (1H; t; J= 7.6 Hz; H-3 of C ring), 6.7 (1H; d; J= 7.5 Hz; H-7 of B ring), 5.1 (2H; S; CH2). Anal. Calcd. for C21H15FN2O (330.36): C,76.35; H, 4.58; F, 5.75; N, 8.48; O, 4.84. Found: C,76.36; H, 4.57; F, 5.79; N, 8.50; O, 4.82.

*1-(4-nitrobenzyl)-3-(phenylimino)indole-2-one (4e)*

M.P. 178-181 oC. Yield: 48.87%. IR (KBr): 1729 (CO), 1600,1461 (Aromatic zone), 1508,1342 (NO2), 1178, 1102,752. MS (ESI): 358 (M+H+). 1H-NMR (500 MHz, CDCl3): 8.22 (2H; d; J= 8.7 Hz; H-3 and H-5 of C ring), 7.55 ( 2H; d; J= 8.7 Hz; H-2 and H-6 of C ring), 7.45 (2H; t; J= 7.5 Hz; H-3 and H-5 of A ring), 7.3 ( 2H; m; H-4 and H-7 of B ring), 7 (2H; d; J= 8.4 Hz; H-2 and H-6 of A ring), 6.8 (1H; t; J= 7.6 Hz; H-4 of A ring), 6.7 (1H; d; J= 7 Hz; H-6 of B ring), 6.65 (1H; d; J= 7.9 Hz; H-5 of B ring), 5.1 (2H; S; CH2). Anal. Calcd. for C21H15N3O3 (357.36): C,70.58; H, 4.23; N, 11.76; O, 13.43.

Found: C,70.59; H, 4.25; N, 11.74; O, 13.40.

*1-(2-nitrobenzyl)-3-(phenylimino)indole-2-one (4f)*

M.P. 174-176 oC. Yield: 43.25%. IR (KBr): 1728 (CO), 1601,1463 (Aromatic zone), 1516,1337 (NO2), 1180, 710. MS(ESI): 358 (M+H+). 1H-NMR (500 MHz, CDCl3): 8.2 (1H; d; J= 8 Hz; H-3 of C ring), 7.62 (1H; t; J= 7.4 Hz; H-4 of B ring), 7.5 (3H; m; H-3 and H-4 and H-5 of A ring), 7.4 (1H; d; J= 7.4 Hz; H-6 of C ring), 7.3 (2H; m; H-5 of B ring and H-5 of C ring), 7.1 (2H; d; J= 7.7 Hz; H-2 and H-6 of A ring), 6.9 (1H; t; J= 7.2 Hz; H-6 of B ring), 6.8 (1H; d; J= 6.8 Hz; H-4 of C ring), 6.7 (1H; d; J= 7.9 Hz; H-7 of B ring), 5.5 (2H; S; CH2). Anal. Calcd. for C21H15N3O3 (357.36): C,70.58; H, 4.23; N, 11.76; O, 13.43. Found: C,70.61; H, 4.23; N, 11.73; O, 13.45.

*1-(4-methylbenzyl)-3-(phenylimino)indole-2-one (4g)*

M.P. 163-166 oC. Yield: 55.25%. IR (KBr): 1728 (CO), 1603,1586,1465 (Aromatic zone), 1356 (CH3), 1186, 1104, 767. MS(ESI): 327(M+H+). 1H-NMR (500 MHz, CDCl3): 7.43 (2H; t; J= 7.6 Hz; H-3 and H-5 of A ring), 7.26 (4H; m; H-4 of A ring and H-6 of B ring and H-2 and H-6 of C ring), 7.15 (2H; d; J= 7.9 Hz; H-2 and H-6 of A ring), 7.02 (2H; d; J= 7.4 Hz; H-3 and H-5 of C ring), 6.75 (1H; d; J= 7.9 Hz; H-4 of B ring), 6.72 (1H; t; J= 7.6 Hz; H-5 of B ring), 6.6 (1H; d; J= 7.6 Hz; H-7 of B ring), 5 (2H; S; CH2), 2.3 (3H; S; CH3). Anal. Calcd. for C22H18N2O (326.39): C,80.96; H, 5.56; N, 8.58; O, 4.90. Found: C,80.94; H, 5.53; N, 8.62; O, 4.93.

*1-(3-methylbenzyl)-3-(phenylimino)indole-2-one (4h)*

M.P. 86-88 oC. Yield: 52.87%IR (KBr): 1728 (CO), 1603,1459 (Aromatic zone), 1340 (CH3), 1100, 761, 689. MS(ESI): 327(M+H+). 1H-NMR (500 MHz, CDCl3): 7.43 (2H; t; J= 7.6 Hz; H-3 and H-5 of A ring), 7.2 (5H; m; H-4 and H-5 and H-6 of B ring and H-2 and H-5 of C ring), 7.1 (1H; d; J= 7.4 Hz; H-4 of C ring), 7.02 (2H; dd; J= 8.4 Hz; J= 1.18 Hz; H-2 and H-6 of A ring), 6.75 (1H; d; J= 7.9 Hz; H-7 of B ring), 6.71 (1H; dt; J= 7.6 Hz; J= 0.8 Hz; H-4 of A ring), 6.62 (1H; d; J= 6.9 Hz; H-6 of C ring), 5 (2H; S; CH2), 2.3 (3H; S; CH3). Anal. Calcd. for C22H18N2O (326.39): C,80.96; H, 5.56; N, 8.58; O, 4.90. Found: C,80.99; H, 5.54; N, 8.59; O, 4.88.

*1-(2-methylbenzyl)-3-(phenylimino)indole-2-one (4i)*

M.P. : 147-150 oC. Yield: 38.37%. IR (KBr): 1732(CO), 1602,1586,1466 (Aromatic zone), 1355 (CH3), 1187, 762. MS (ESI): 327(M+H+). 1H-NMR (500 MHz, CDCl3): 7.5 (2H; m; H-4 and H-7 of B ring), 7.2 (6H; m; H-3 and H-5 of A ring; H-5 and H-6 of B ring; H-4 and H-5 of C ring), 7.1 (2H; d; J= 7.5 Hz; H-2 and H-6 of A ring), 6.8 (1H; t; J= 7.4 Hz; H-4 of A ring), 6.7 (1H; d; J= 7.9 Hz; H-6 of C ring), 6.6 (1H; d; J= 7.9 Hz; H-3 of C ring), 5 (2H; S; CH2), 2.5 (3H; S; CH3). Anal. Calcd. for C22H18N2O (326.39): C,80.96; H, 5.56; N, 8.58; O, 4.90. Found: C,80.95; H, 5.55; N, 8.57; O, 4.89.

*1-(4-methoxybenzyl)-3-(phenylimino)indole-2-one (4j)*

M.P. : 119-122 oC. Yield: 18.25%. IR (KBr): 1742(CO), 1621,1525,1477 (Aromatic zone), 1162, 783. MS(ESI): 343(M+H+). 1H-NMR (500 MHz, CDCl3): 7.4 (1H; d; J= 8.5 Hz; H-4 of B ring), 7.3 (1H; d; J= 8.6 Hz; H-5 of B ring), 7.2 (7H; m; H-2 and H-3 and H-4 and H-5 and H-6 of A ring; H-2 and H-6 of C ring), 7.1 (1H; H-6 of B ring), 7 (1H; d; J= 8.5 Hz; H-7 of B ring), 6.9 (2H; d; J= 8.6 Hz; H-3 and H-5 of C ring), 5 (2H; S; CH2), 3.8 (3H; S; OCH3). Anal. Calcd. for C22H18N2O2 (342.39): C,77.17; H, 5.30; N, 8.18; O, 9.35. Found: C,77.19; H, 5.33; N, 8.21; O, 9.34.

*1-(3-methoxybenzyl)-3-(phenylimino)indole-2-one (4k)*

M.P. : 114-116 oC. Yield: 24.62%. IR (KBr): 1730(CO), 1605,1577,1461 (Aromatic zone), 1342 (CH3), 1148, 1038, 765, 692. MS(ESI): 343(M+H+). 1H-NMR (500 MHz, CDCl3): 7.43 (2H; t; J= 7.5 Hz; H-3 and H-5 of A ring), 7.2 (3H; m; H-4 and H-5 and H-6 of B ring), 7 (2H; d; J= 8.5 Hz; H-2 and H-6 of A ring), 6.8 (1H; d; J= 8 Hz; H-7 of B ring), 6.75 (1H; d; J= 7.9 Hz; H-6 of C ring), 6.71 (1H; t; J= 7.6 Hz; H-4 of A ring), 6.6 (1H; d; J= 6.9 Hz; H-4 of C ring), 5 (2H; S; CH2), 2.3 (3H; S; OCH3);(Mixed isomers). Anal. Calcd. for C22H18N2O2 (342.39): C,77.17; H, 5.30; N, 8.18; O, 9.35. Found: C,77.20; H, 5.28; N, 8.15; O, 9.37.

*Biological activity*

*In-vitro evaluation of antiplatelet aggregation activity*

The antiplatelet aggregation activity of the new derivatives were measured using human plasma on an APACT 4004 aggregometer according to the method described before (22). Fresh blood samples were obtained from non-smoker healthy volunteers with negative history of drug consumption up to 15 days prior to the test. Platelet-rich plasma (PRP) was obtained from whole blood collected in sodium citrate (9:1 by volume) upon centrifugation at 1000 rpm for 8 min. The remaining was centrifuged at 3000 rpm for 15 min and PPP was collected from the above layer which was used as the test blank. The platelet count was adjusted to 250000 plts/mL by diluting PRP with appropriate amount of PPP. To PRP samples, test compounds previously dissolved in ethylenglycol monomethyl ether were added and samples were incubated for 5 min at 37 °C. Then ADP (5 μM) or arachidonic acid 1.35 (mM) was added and platelet shape change and aggregation were monitored for 5 min. Ethylenglycol monomethyl ether (0.5% v/v) was used as negative control and indomethacin and aspirin as standard drugs. Platelet aggregation inhibition (%) was calculated by the following formula:

Inhibition % = [1 – (D/S)] × 100

Where D = platelet aggregation in the presence of test compounds and S = platelet aggregation in the presence of solvent.

Compounds were thus screened at the primary concentration of 100 μM and those that exhibited higher than 50% inhibitory activity, were further diluted to calculate IC50.

## Results and Discussion

*Chemistry*

**Scheme 1:**
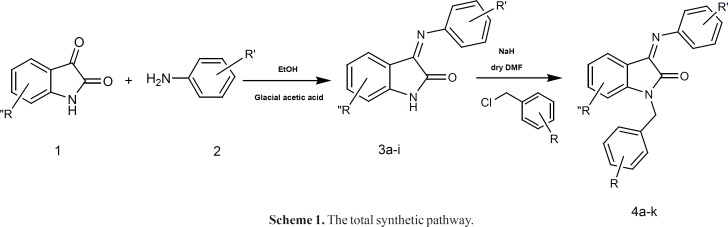
The total synthetic pathway

The synthetic pathway is disclosed in scheme 1. The appropriate aniline derivative was reacted with isatin (or substituted isatin) to form the desired imine structure. The so formed compound was then activated using NaH and reacted with the proper substituted benzyl halides.

*Biology*

The *in-vitro *antiplatelet activity of all the synthesized compounds was assayed on human platelet rich plasma (PRP) by using the Born’s reported turbidimetric method (23). ADP and Arachidonic acid (AA) were employed as inducers of platelet aggregation, and indomethacin and aspirin were used as the positive controls. IC50 values were calculated for compound that inhibited platelet aggregation more than 90%. IC50 was defined as the concentration of the test compound that inhibits platelet aggregation by 50%.

The antiplatelet aggregation activity of the derivatives is listed in Table 1. Data show that the majority of the derivatives inhibited AA-induced platelet aggregation more effectively than the aggregation induced by ADP and some compounds showed inhibitory effects comparable to Indomethacin. Among the tested compounds, derivatives 4a, 4c, 4d, 4f-i and 4k showed high IC50 values and 1-(2-nitro benzyl)-3-(phenyl imino)indole-2-one was the most potent compound with IC50 value of 3.4 μM against aggregation induced by AA. Based on the activity data, it could be suggested that the most active compounds were among the most lipophilic structures. The presence of substitution on para position of benzyl ring decreased the antiplatelet activity (compounds 4b, 4e and 4j).This may be due to the hindrance caused by para substituent in ligand-receptor interaction. However the high activity of compound 4 g (IC50 = 14.5 mM) which has a 4-methylbenzyl substituent on position 1, is not consistent with this hypothesis and suggest that other parameters such as lipophilic and electronic properties of the aromatic ring may possibly contribute to the activity of the compounds.

**Table 1:** Antiplatelet aggregation activity of the synthesized derivatives

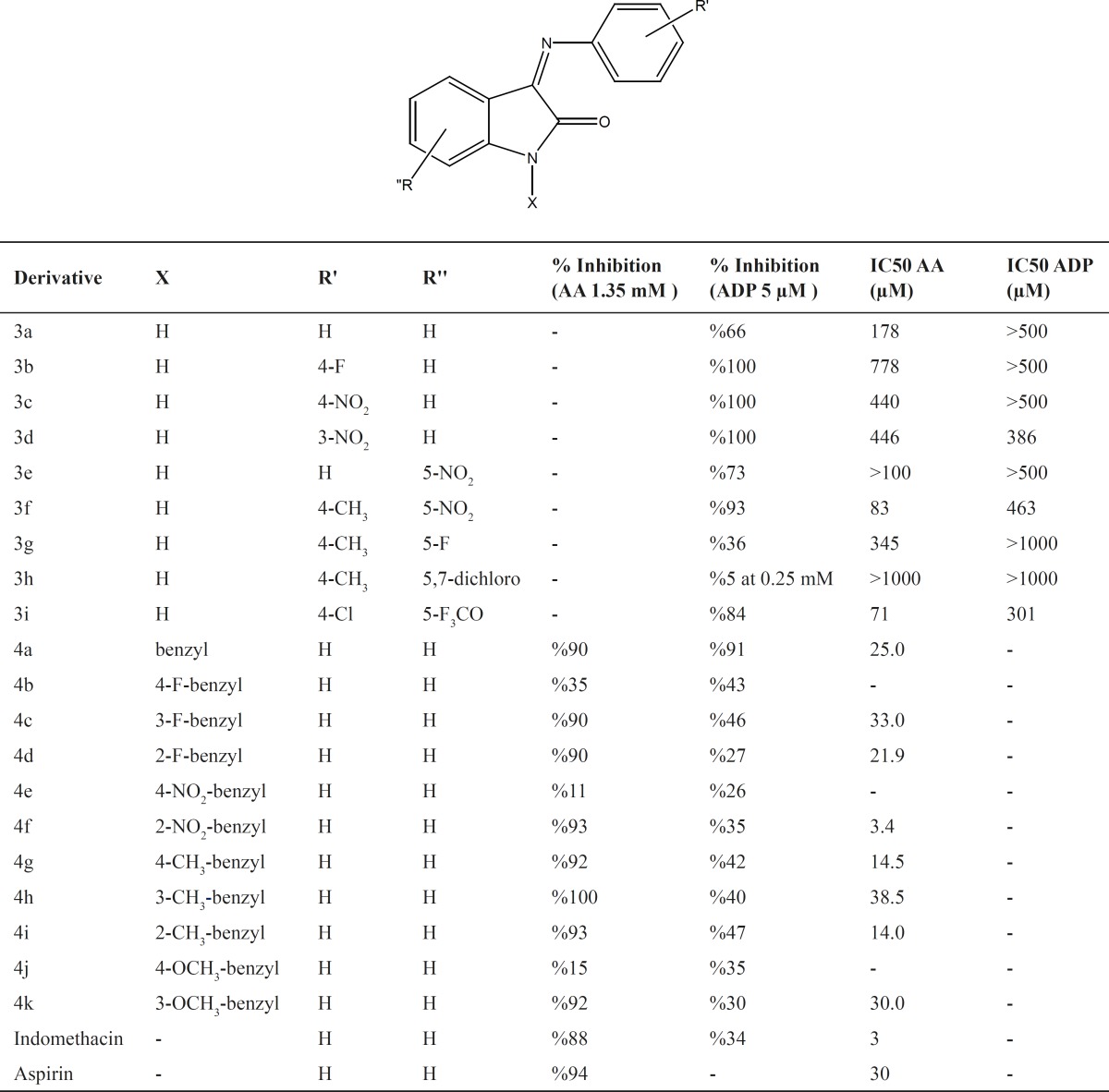

In order to further verify the contribution of benzyl substituent as a pharmacophore in antiplatelet activity of indole derivatives, a few other derivatives were prepared which in their structure the benzyl ring is absent but other substituents are present either on phenylimino ring or on indole ring itself (compounds 3a-i), Less potent than the weakest N-benzylated analog (compound 4h with IC50 value of 38.5 μM). This finding reemphasizes the significance of N-benzylation of indole ring for optimum antiplatelet aggregation activity.

## Conclusion

In the present study a series of N-benzyl substituted indole derivatives have been synthesized and their antipla telet activities were assessed against ADP and AA-induced platelet aggregation in human plasma. The tested derivatives selectively inhibited platelet aggregation induced by AA with very good IC50 values. Among them, compound 4f with IC50 of 3.4 μM proved to be the most potent derivative of the series.
